# Myeloid cell leukemia-1 (*Mc1*-*1*) is a candidate target gene of hypoxia-inducible factor-1 (*HIF*-*1*) in the testis

**DOI:** 10.1186/1477-7827-10-104

**Published:** 2012-12-05

**Authors:** Michael A Palladino, Anoop Shah, Rebecca Tyson, Jaclyn Horvath, Christine Dugan, Marie Karpodinis

**Affiliations:** 1 Department of Biology, Monmouth University, 400 Cedar Avenue, West Long Branch, NJ, 07764, USA

**Keywords:** Apoptosis, Torsion, Ischemia, Leydig cells

## Abstract

**Background:**

Spermatic cord torsion can lead to testis ischemia (I) and subsequent ischemia-reperfusion (I/R) causing germ cell-specific apoptosis. Previously, we demonstrated that the hypoxia-inducible factor-1 (HIF-1) transcription factor, a key regulator of physiological responses to hypoxia, is abundant in Leydig cells in normoxic and ischemic testes. We hypothesize that testicular HIF-1 activates the expression of antiapoptotic target genes to protect Leydig cells from apoptosis. *In silico* analysis of testis genes containing a consensus hypoxia response element (HRE, 5’-RCGTG-3’) identified myeloid cell leukemia-1 (*Mcl*-*1*) as a potential HIF-1 target gene. The purpose of this study was to determine whether HIF-1 shows DNA-binding activity in normoxic and ischemic testes and whether *Mcl*-*1* is a target gene of testicular HIF-1.

**Methods:**

The testicular HIF-1 DNA-binding capacity was analyzed *in vitro* using a quantitative enzyme-linked immunosorbent assay (ELISA) and electrophoretic mobility shift assays (EMSA). MCL-1 protein expression was evaluated by immunoblot analysis and immunohistochemistry. The binding of testicular HIF-1 to the *Mcl*-*1* gene was examined via chromatin immunoprecipitation (ChIP) analysis.

**Results:**

The ELISA and EMSA assays demonstrated that testicular HIF-1 from normoxic and ischemic testes binds DNA equally strongly, suggesting physiological roles for HIF-1 in the normoxic testis, unlike most tissues in which HIF-1 is degraded under normoxic conditions and is only activated by hypoxia. MCL-1 protein was determined to be abundant in both normoxic and ischemic testes and expressed in Leydig cells. In a pattern identical to that of HIF-1 expression, the steady-state levels of MCL-1 were not significantly affected by I or I/R and MCL-1 co-localized with HIF-1α in Leydig cells. Chromatin immunoprecipitation (ChIP) analysis using a HIF-1 antibody revealed sequences enriched for the *Mcl*-*1* promoter.

**Conclusions:**

The results demonstrated that, unlike what is observed in most tissues, HIF-1 displays DNA-binding activity in both normoxic and ischemic testes, and *Mcl*-*1* may be a key target gene of testicular HIF-1 with potential roles in the antiapoptotic protection of Leydig cells.

## Background

Hypoxia-inducible factor-1 (HIF-1), often described as a “master” regulator of hypoxic responses in mammalian cells, is a well-characterized transcription factor known to activate over 100 different hypoxia-sensitive genes involved in a range of metabolic processes including anti- and proapoptotic responses to ischemia and hypoxia [1,2]. Thus, HIF-1 has been widely studied due to its implication in a range of disease conditions and ischemic processes of clinical importance [3,4].

We are interested in studying HIF in the testis in part because clinical conditions such as testicular torsion (spermatic cord torsion), varicocoele and hypobaric hypoxia can cause hypoxic and ischemic injury of the testis [5-7]. Germ cell-specific apoptosis [8], activation of inflammatory pathways [9], lipid peroxidation [7] and alterations in testis protein profiles [10] are among the many effects of hypoxia on the testis that have been widely-studied, although Leydig cells and Sertoli cells appear to be generally resistant to ischemia and hypoxia-induced apoptosis [8].

HIF-1 can have antiapoptotic or apoptotic effects on cells depending on the extent and duration of the oxygen debt [11]. We hypothesize that HIF-1 plays important roles in mediating antiapoptotic responses in the testis and may protect Leydig cells from apoptosis. Previously we demonstrated that HIF-1α mRNA and protein is abundantly expressed by Leydig cells in the rat testis [12,13]. These studies revealed that, unlike many tissues in which HIF-1α expression is induced by hypoxia, HIF-1 is constitutively expressed in the normoxic testis and the levels of testicular HIF-1 are unaffected following ischemia and hypoxia caused by testicular torsion and reperfusion [12]. HIF-1α is also expressed in Leydig cells in the normoxic mouse testis [14].

A body of work is emerging that demonstrates that HIF-1α may be functionally active in normoxic tissues [15]. Measurements of the physiological oxygen tension in the testis indicate that testicular pO_2_ levels are likely low, bordering on hypoxia [16-18] and it has been suggested that other factors such as reactive oxygen species render the testis hypoxic even under presumptive normoxic conditions. If the testis is indeed a hypoxic tissue, then this may explain the constitutive expression of HIF-1 in the testis. However, little is known about HIF-1 target genes in the testis, and this area of research is of interest to investigators seeking to understand the cellular and molecular responses of Leydig cells to changes in the oxygen microenvironments in the testis. Lysiak et al. showed that in the mouse testis, HIF-1α regulates the expression of the 3β-hydroxysteroid dehydrogenase type I (*Hsd3b1*) gene [14].

In the present study, we wanted to determine if testicular HIF-1 is active and capable of binding DNA under normoxic and hypoxic conditions. Additionally, we propose that another possible target for testicular HIF-1 is the myeloid cell leukemia-1 (*Mcl*-1) gene, an antiapoptotic member of the Bcl-2 family, which was first discovered in the human ML-1 myeloid leukemia cell line [19]. We used in vitro DNA-binding assays (ELISA and EMSA) to demonstrate that testicular HIF-1 from normoxic and ischemic testes displays DNA-binding activity. When we examined the expression of *Mcl1* as a potential antiapoptotic target gene of testicular HIF-1, MCL-1 protein was determined to be abundant in Leydig cells in both normoxic and ischemic testes. ChiP analysis using an HIF-1 antibody revealed sequences enriched for Mcl-1 promoter, providing evidence for Mcl-1 as a key target gene of testicular HIF-1 with potential roles in antiapoptotic protection of Leydig cells.

## Methods

### Animals and surgical procedures

Adult male, retired-breeder Sprague–Dawley rats (550–650 g) purchased from Charles River Laboratories (Stone Ridge, NY) were housed under controlled conditions of temperature and humidity on a twelve-hour light/dark cycle. Animal care and surgical procedures were carried out following guidelines established by the National Institutes of Health (NIH) Public Health Service Policy and the Monmouth University Institutional Animal Care and Use Committee (IACUC).

Animals were anesthetized using injections of sodium pentobarbital (Sigma-Aldrich, St. Louis, MO) at a concentration of 75 mg of pentobarbital per kg of body weight. Torsion surgeries were performed via a midline laparotomy using sterile procedures and with animals maintained on warming pads to prevent hypothermia as previously described [8,13]. Torsion was induced unilaterally to create an experimental state of ischemia. Following an abdominal incision, the gubernaculum was cut and the testis and epididymis were exposed through the laparatomy incision. The connective tissue holding the testis to the epididymis was separated and the testis rotated 720° clockwise. Then, the testis and epididymis were returned to the scrotum, the laparatomy incision closed and the testis maintained in torsion. In reperfusion experiments, each torsed testis was detorsed and the testicular and scrotal stumps of the divided gubernaculum were sutured together to hold the testis in place for the duration of the reperfuson treatment phase and the testis and epididymis were returned to the scrotum.

Contralateral, sham-treated testes served as controls. The surgeries were carried out on alternating sides so that sham or ischemia induction was performed in right and left testes for all time points. Each animal had a sham testis and an ischemic testis. Animals were maintained under anesthesia for the duration of the surgical treatment.

The treatment groups involved the following conditions (*n* = 3–6 animals/group): 1 hour or 6 hours of ischemia (I), or 1 hour of ischemia followed by 4 hours of reperfusion (1h/4h I/R). At the conclusion of the treatment, euthanasia was performed via carbon dioxide asphyxiation in accordance with the Report of the American Veterinary Medical Association (AVMA) Panel on Euthanasia (2000). The testes were excised, then frozen in liquid nitrogen and stored at −70°C prior to RNA or protein isolation. Apoptosis of germ cells in the testes following I and I/R was evaluated via terminal deoxynucleotidyltransferase mediated dUTP-biotin nick end labelling (TUNEL) assays to validate cell damage. These data were previously reported in Supplemental Data of Palladino et al. [12] and demonstrated an increase in germ cell-specific apoptosis following I and I/R, but no statistically significant changes in apoptosis of Leydig cells after I and I/R.

### Isolation of cytoplasmic and nuclear proteins

Fresh or frozen tissue samples were placed on ice and homogenized in 3 volumes of lysis buffer composed of 10 mM Tris–HCl (pH 7.5), 1.5 mM MgCl_2_, and 10 mM KCl with freshly added 1 mM dithiothreitol (DTT), 1 mM Na_3_VO_4_, and 10 mM protease inhibitor mix (P8340; Sigma-Aldrich, St. Louis, MO). The homogenate was centrifuged at 3,500 rpm at 4°C for 5 minutes and the resulting supernatant was saved as the cytoplasmic fraction and stored at −80°C. To isolate nuclear proteins, the pellet obtained from the initial centrifugation step was resuspended in 0.42 M KCl, 20 mM Tris–HCl (pH 7.5), 1.5 mM MgCl_2_, and 20% glycerol, with freshly added 1 mM dithiothreitol (DTT), 1 mM Na_3_VO_4_, and protease inhibitor cocktail (P8340; Sigma-Aldrich), mixed at 150 rpm at 4°C for 30 minutes to liberate proteins from nuclei and then centrifuged at 13,500 rpm for 30 minutes at 4°C. The resulting supernatant containing nuclear proteins was removed and stored at −80°C. Protein concentrations were determined via the Bradford assay (Bio-Rad, Hercules, CA).

### Enzyme-linked immunosorbent assay (ELISA) to detect HIF-1 DNA binding

Testicular HIF-1 DNA binding was determined using the Active Motif TransAM™ HIF-1 Transcription Factor Assay Kit (Active Motif, Carlsbad, CA) following the manufacturer’s instructions. In this assay, wells are pre-coated with oligonucleotides containing the consensus hypoxia response element (HRE: 5’-TACGTGCT-3’, consensus sequence for the HRE underlined) from the human *EPO* gene and HIF-1 DNA binding of protein extracts is determined by incubating 10 μg aliquots of nuclear proteins in the wells for 1 hour at room temperature. Protein extracts from CoCl_2_-treated hypoxic COS-7 cells (Active Motif) were used as positive controls for HIF-1 binding. After several washing steps, 100 μl of a goat anti-HIF-1α primary antibody (AF1935; R&D Systems, Inc., Minneapolis, MN) was added, followed by incubation for 1 hour at room temperature and detection with an anti-IgG-horseradish peroxidase (HRP)-conjugated secondary antibody. Colorimetric changes were determined via spectrophotometric measurement of the absorbance at 450 nm, with a reference reading obtained at 655 nm and a background calculation deducted from each well. The absorbance readings were quantitated as a fold increase of the negative control. Samples were run in triplicate using protein extracts isolated from testes treated for different times under I or I/R (*n* = 3–6 animals for each treatment group) and the statistical significance of results was determined via Analysis of Variance (ANOVA).

### Electrophoretic mobility shift assays (EMSA)

Electrophoretic mobility shift assays were performed using the Thermo Scientific LightShift® Chemiluminescent EMSA kit according to the manufacturer’s instructions (Thermo Scientific, Rockford, IL). The reactions were carried out in 20 μl volumes with 5 μg of a nuclear or cytoplasmic protein extract from the testis incubated with 10× binding buffer, 1 μg/μl poly (dI·dC), 50% glycerol, 1% NP-40, 1M KCl, 100 mM MgCl_2_, and 200 mM EDTA and biotin-labelled EMSA oligonucleotides for 20 minutes at room temperature. Epstein-Barr Nuclear Antigen (EBNA) and biotin-labelled double-stranded target DNA was included as a positive control for the EMSA. A double-stranded HIF-1 oligonucleotide containing the consensus HRE (5’-TCTGTACGTGACCACACTCACCTC-3’, consensus sequence for the HRE underlined; sc-2625, Santa Cruz Biotechnology, Santa Cruz, CA) and mutant oligonucleotides (sc-2626; Santa Cruz Biotechnology) with an AAA substitution in place of the CGT in the consensus site were used for EMSA.

Terminal deoxynucleotidyl transferase (TdT) was employed to catalyze the biotin labelling of the oligonucleotides used for EMSA according to the manufacturer’s instructions (Pierce Biotin 3’ End DNA Labelling Kit). Labelling reactions with a 50 μl volume containing the appropriate oligonucleotides were incubated for 30 minutes at 37°C in ultrapure water, 5X TdT Reaction Buffer, Biotin-11 UTP, and diluted TdT. To stop each reaction, 0.2 M EDTA was added and 24:1 chloroform: isoamyl alcohol extraction performed to remove protein from labelled oligonucleotide. Labelled oligonucleotides were stored at −20°C.

The EMSA reactions were separated using 6% polyacrylamide TBE PAGEr® Gold gels (Lonza, Rockland, ME) electrophoresed in 0.5× (0.89 M) Tris-borate-EDTA buffer (Lonza AccuGENE® 10× TBE Buffer). Binding reactions were transferred onto nylon membrane (Thermo Scientific Biodyne® B) which were then cross-linked at 120 mJ/cm^2^ for 60 seconds using a UV-light cross-linker instrument. The blots were blocked with blocking buffer, incubated with a 1:300 dilution of a stabilized streptavidin-HRP conjugate for 15 minutes at room temperature and then reacted with luminal/enhancer solution (Pierce Chemiluminescent Nucleic Acid Detection Module). The blots were exposed to x-ray film (BioMax ML; Kodak, Rochester, NY) for 2–5 minutes, or digital images were captured using a ChemiDoc™ XRS+ Molecular Imager with Image Lab™ Software (Bio-Rad).

For competition experiments, an excess of the unlabeled HIF-1α consensus oligonucleotide was used to demonstrate competition for DNA binding between biotin-labelled and unlabeled oligonucleotides. In supershift experiments, the specificity of HIF-1 DNA binding was demonstrated on 4-20% polyacrylamide TBE PAGEr® Gold gels which were electrophoresed in 0.5× Tris-borate-EDTA (Lonza). Goat anti-human polyclonal antibodies C-19 and Y-15 (sc-8711 and sc-12542 respectively; Santa Cruz Biotechnology) were used to perform supershift experiments. All EMSA experiments were replicated a minimum of 5 times with independent samples (*n*=3-6 animals for each treatment group). Representative results are presented.

### Immunoblot analysis

Immunoblot analysis was performed following denaturing sodium dodecyl sulfate (SDS) polyacrylamide gel electrophoresis (PAGE) of nuclear and cytoplasmic samples in 7.5% or 10% PAGEr® Gold Tris-Glycine polyacrylamide gels (Lonza, Rockland, ME). Tissue extracts obtained from COS-7 cells (Active Motif, Carlsbad, CA), rat PC-12 pheochromocytoma (Active Motif), and the BJAB human B cell lymphoma line (Santa Cruz Biotechnology, Santa Cruz, CA) were included as positive controls for detecting HIF-1. Seventy-five microgram aliquots of protein samples were heated for 5 minutes at 95°C prior to loading and then electrophoresed at 150 V for approximately 90 minutes. Proteins were electroblotted onto Trans-Blot nitrocellulose membranes (Bio-Rad), and Ponceau S staining (0.0005% in 1% acetic acid) was used to confirm protein transfer. The blots were blocked in 1× Western Wash (50 mM Tris, pH 7.6, 30 mM NaCl, 0.001% Tween 20) containing 5% nonfat dry milk under gentle agitation at room temperature for 30 minutes. For the detection of MCL-1, the blots were incubated overnight with gentle agitation at 4°C in a 1:1,000 dilution of an Mcl-1 primary antibody (rabbit polyclonal IgG;C. sc819, Santa Cruz Biotechnology) in 1× Western Wash and 5% nonfat dry milk.

After 3 washes with 1× Western Wash, the blots were incubated with an HRP-conjugated secondary antibody (goat anti-rabbit IgG; sc2054) at a 1:20,000 dilution in 1× Western Wash with 5% nonfat dry milk for 1 hour at room temperature under gentle agitation. Following 3 washes with 1×7 Western Wash, the blots were developed via enhanced chemiluminescence in SuperSignal West Pico or Femto substrate (Pierce, Rockford, IL) and exposed to X-ray film (Biomax ML).

The blots were stripped of bound antibody by incubation in Restore Buffer (Pierce) at 37°C for 15 minutes and then washed in 1× Western Wash for 5 minutes, prior to blocking and reprobing. The blots were probed for activating transcription factor-2 (ATF-2) using a 1:1,000 dilution of an anti-ATF-2 antibody (rabbit polyclonal IgG; sc-6233) to detect ATF-2 as a hypoxia-independent loading control for protein quantification. Negative control experiments to determine antibody specificity were carried out by incubating the blots with pre-immune sera, followed by incubation with secondary antibodies. Mcl-1 protein levels were normalized to ATF-2 as a loading control. Immunoblot data were analyzed by one-way ANOVA and the results considered significantly different at *P*< 0.05.

Immunoblot analysis was conducted to determine the presence of MCL-1 in cultured Leydig cells. Cultured cells were provided by the Hardy lab of the Population Council at the Center for Biomedical Research (New York, NY). Briefly, Leydig cells were purified from 325 to 350 g Sprague–Dawley rats using a previously described collagenase dispersal and sedimentation approach [20]. Typically, 6 animals were required to yield 10 to 20 million adult Leydig cells. A hemocytometer was used to estimate cell yields and the percentage of purity was assessed via histochemical staining to detect 3β-hydroxysteroid dehydrogenase with etiocholan-3β-ol-17-one as the enzyme substrate. The adult Leydig cell preparations used for these experiments were over 95% pure. Equal number of cells were allocated to T-25 flasks and cultured in buffered Dulbecco’s modified Eagle’s medium: F12 culture medium supplemented with ovine luteinizing hormone (0.1 ng/mL) and lipoproteins (Lipimate; Hyclone Logan, Utah). Fresh isolates of cells were used for isolating MCL protein, or cells were cultured at 34° C for 20 hours in an incubator containing either a 5% O_2_/5% CO_2_ atmosphere or a 21% O_2_/5% CO_2_ atmosphere. Five separate replicates were performed for each of the culture conditions in these experiments. Leydig cells were collected from the flasks using trypsin, centrifuged and frozen in liquid nitrogen as cell pellets for subsequent MCL protein isolation.The applied oxygen concentrations were chosen to mimic hypoxic and normoxic conditions, respectively.

Cytoplasmic extracts were isolated and separated on 10% Tris-glycine polyacrylamide gels via SDS-PAGE and immunoprobing procedures were carried out as described above. The immunoblots were run with the total protein aliquots isolated from equivalent numbers of cells (1 million cells) for each treatment group.

### Immunocytochemistry (IHC)

To determine which cell types in the testis express Mcl-1, tissue sections were prepared for immunocytochemistry. Tissues were placed in 10% (w/v) neutral-buffered formalin (Richard-Allan Scientific, Kalamazoo, MI) overnight prior to paraffin embedding. The tissue samples were then dehydrated in three successive changes of 70%, 95% and 100% ethanol then transferred through three changes of xylene and incubated overnight at 60°C in a 1:1 xylene/molten paraffin mixture. Finally, the samples were incubated at 60°C in fresh molten paraffin for 1 hour through a total of 3 changes and then embedded in paraffin. Paraffin sections were cut at a thickness of 5 μm and mounted on glass slides (Superfrost® Plus; VWR).

The sections were deparaffinized in xylene and hydrated through a series of graded ethanol solutions and then rinsed in 1× phosphate-buffered saline (PBS) solution for 5 minutes to complete the hydration steps. Then, the slides were covered in antigen unmasking solution (Vector Laboratories, Inc., Burlingame, CA), microwaved on high for 10 minutes and cooled for 1 hour at room temperature. The slides were next rinsed in 1× PBS for 5 minutes and quenched in 0.5% (v/v) H_2_O_2_/methanol for 30 minutes, then washed in 1× PBS for 5 minutes, placed into a humidified chamber, and incubated with blocking serum for 20 minutes using serum from the species of origin of the secondary antibody. A 1:100 dilution of an Mcl-1 primary antibody (rabbit polyclonal IgG; sc819) in 1× PBS was added to the testis samples as well as slides containing sections of spleen as a positive control, and the slides incubated in a humidified chamber overnight at 4°C. Negative control sections were incubated in the absence of primary antibody.

The slides were washed in 1× PBS for 5 minutes with a total of 3 changes. The slides were then incubated in biotinylated secondary antibody (goat anti-rabbit IgG; sc2054) at a 1:200 dilution in 1× PBS combined with Santa Cruz blocking serum for 45 minutes in a humidified chamber at room temperature. Next, the slides were washed in 1× PBS for 5 minutes through a total of 3 changes and incubated in the Santa Cruz AB (avidin-biotin) enzyme reagent for 30 minutes at room temperature. The slides were subsequently washed in 1× PBS through a total of 3 changes, and sections were incubated with diaminobenzidine/peroxidase substrate for 10 minutes at room temperature followed by washing with dH_2_O prior to counterstaining with hematoxylin. Finally, the sections were dehydrated in a graded series of ethanol solutions, placed in xylene, and cover slips were mounted using Permount (Fisher Scientific, Pittsburgh, PA). The slides were examined under a Nikon Eclipse 50i light microscope (Nikon Instruments, Inc., Melville, NY) and photographed using a DS-L1 digital camera.

### Chromatin immunoprecipitation (ChIP) assays

ChIP assays were carried out with the Active Motif ChIP-IT™ Express kit as per protocols outlined in the manufacturer’s instruction manual. In place of cultured cells, a 0.50 g sample of freshly excised testis from sham and surgically treated animals was used to isolate chromatin complexes. The tissue samples were incubated in 20 ml of fixation solution (37% formaldehyde; Dulbecco’s modified Eagle’s medium) under gentle agitation for 10 minutes at room temperature, then washed with ice-cold 1× PBS. Fixation was stopped by adding 10 ml of a glycine-1× PBS stop-fix solution followed by swirling and rocking at room temperature for 5 minutes. The samples were washed with 1× ice-cold PBS and cells were pelleted by centrifugation at 2,500 rpm for 10 minutes at 4°C. Finally, 1 μl of 100 mM phenylmethylsulfonylfluoride (PMSF) and 1 μl protease inhibitor cocktail (PIC) were added to the cell pellets, which were then stored at −80°C.

For ChIP assays, the pellets were subsequently thawed in 1 ml of ice-cold lysis buffer (supplemented with 5 μl PIC + 5 μl PMSF) and incubated on ice for 30 minutes. Cell nuclei were released by homogenizing the cell pellets using an ice-cold glass dounce homogenizer followed by centrifugation at 5,000 rpm for 10 minutes at 4°C to pellet chromatin. The pellets were resuspended in 1.0 ml of digestion buffer pre-warmed at 37°C and supplemented with 5 μl PIC + 5 μl PMSF.

Chromatin DNA was enzymatically sheared using DNase (200U/ml) and optimal enzymatic shearing conditions determined by placing 50 μl aliquots of chromatin in digestion buffer that had been pre-warmed at 37°C for 5 minutes and adding a working stock of an enzymatic shearing cocktail to the chromatin, followed by incubation for 5, 10 or 15 minutes. The reactions were stopped by adding 1 μl of ice-cold 0.5 M EDTA followed by chilling on ice for 10 minutes. Sheared chromatin samples were then centrifuged at 10,000 rpm for 10 minutes at 4°C and stored at −80°C. Proteinase K-treated chromatin samples were electrophoresed through 1% agarose gels prepared in 1× sodium boric (SB) acid buffer (Faster Better Media, LLC, Baltimore, MD) [21] containing SYBR® Safe (Invitrogen) and visualized to determine the size range of the sheared DNA and optimum shearing conditions.

A magnetic bead system was employed to capture HIF-1/chromatin complexes. Briefly reaction tubes were loaded with Protein G magnetic beads, ChIP Buffer, sheared chromatin, PIC, and 100 μg of a HIF-1α primary antibody (AF1935; R&D Systems, Inc., Minneapolis, MN) diluted in 3 μl of 1× PBS in 100 μl reaction volumes. Chromatin in positive control samples was incubated with a primary antibody for RNA polymerase II (ChIP-IT™ Express Enzymatic, Active Motif), and negative control samples were incubated with a nonspecific IgG antibody or polyclonal antibody to β-actin. The reactions were incubated on a rolling shaker for 4 hours at 4°C, and the tubes were then placed on a magnetic stand to pellet the beads on the side of the tube. The supernatant was discarded and the beads were washed three times with ChIP buffers and reverse cross-linking buffer to elute chromatin.

Polymerase chain reaction (PCR) amplification was employed to identify ChIP-enriched sequences. The PCR master mix consisted of the following components: 196 μl of diethylpyrocarbonate-treated H_2_O, 50 μl 10× PCR buffer, 50 μl 10× loading dye, 20 μl of a dNTP mixture, 4 μl of *Taq* polymerase, and 80 μl of dH_2_O (for control PCR sample only) or 40 μl forward/40 μl reverse primers (either for β-*actin* or for *Mcl*-1). The rat β-*actin* primer came from the ChIP-IT™ Control Kit (Active Motif). *Mcl*-*1* forward (5’-ACTTGAGGCCATGAGTTCGAGACCA-5’) and reverse (5’-CTCCACTTCCCACGTTCAGACGATT-3’) primers were designed based on the rat *Mcl*-*1* promoter sequence (AF147742.1) spanning the HRE portion of the promoter and were purchased from MWG Biotech Inc. (High Point, NC).

Aliquots of chromatin samples were incubated in PCR mix containing either β-*Actin* primers or *Mcl*-1 primers, and the samples were amplified using the following thermocycling conditions: 36 cycles of 94°C for 20 seconds, 59°C for 30 seconds, and 72°C for 30 seconds. Analysis of the obtained PCR products was carried out via electrophoresis of 8 μl aliquots of each amplified sample on 2% agarose gels prepared in 1× SB buffer containing SYBR® Safe. The PCR products were cloned into pGEM-T Easy plasmid vectors (Promega Corp., Madison, WI) and sequences of enriched DNA were confirmed by cycle sequencing using a LI-COR 4300L DNA sequencer (LI-COR Biosciences, Lincoln, NE) and BLAST (Basic Local Alignment Search Tool analysis (Altschul, et al., 1990).

## Results

### Testicular HIF-1 from normoxic and ischemic testis displays DNA-binding activity

We previously demonstrated [12] that unlike many other tissues in which HIF-1 accumulates only after hypoxia, HIF-1 is abundant in normoxic testes. This result suggested potentially novel roles for HIF-1 in the testis. Thus, we sought to determine if testicular HIF-1 is capable of DNA binding in the normoxic testis and to determine if HIF-1 DNA binding is affected by the ischemic conditions that cause testis hypoxia. ELISA and EMSAs to detect HIF-1 DNA binding were used to examine the testicular HIF-1 DNA-binding ability.

**Figure 1:**
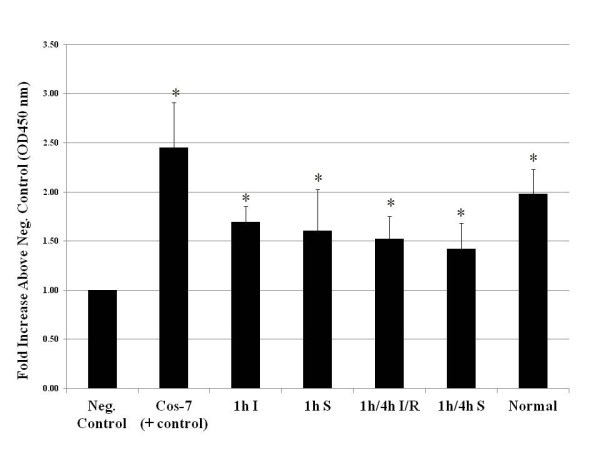
**Testicular HIF**-**1 displays DNA**-**binding activity by ELISA.** Nuclear protein extracts from ischemic (I) testes, sham-treated (S) testes, and testes subjected to ischemia-reperfusion (I/R) demonstrating *in vitro* binding to the HIF HRE in ELISA experiments. Absorbance readings were quantitated as the fold increase over negative control values. COS-7 protein extracts were included as positive controls for HIF-1 DNA binding. The HIF-1 binding in experimental samples was significantly higher than that in negative control samples (*ANOVA, p<0.05), but there were no statistically significant differences in binding activity among the experimental samples.

The results of the HIF-1 DNA-binding ELISA demonstrated binding of testicular HIF-1 to HRE sequences contained in microwell plates (Figure 1). The HIF-1 DNA-binding activities in testes protein samples from sham, 1 h I and 1h/4h I/R experiments were significantly higher than in negative control samples, but there was no significant difference in the binding activities between experimental samples (ANOVA, p<0.05), indicating that the normoxic and ischemic samples showed similar levels of testicular HIF-1 DNA-binding activity.

### Electrophoretic mobility shift experiments demonstrate testicular HIF-1 binding

**Figure 2:**
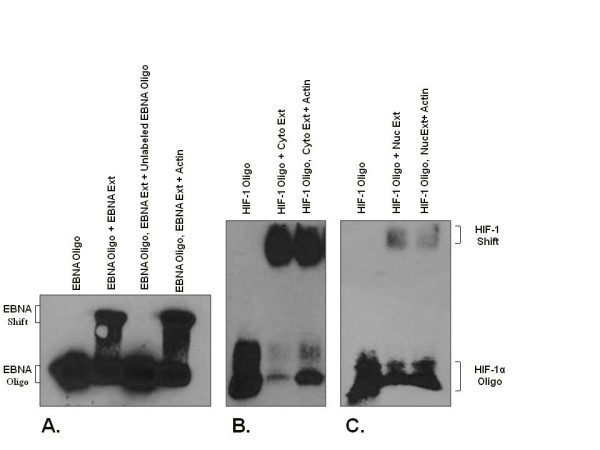
**Electrophoretic Mobility Shift Assays** (**EMSA**) **for testicular HIF**-**1 binding.** The DNA-binding activity of testicular HIF-1 was determined in EMSA experiments. (**A**) Results of positive control experiment using biotin-labelled oligonucleotides for Epstein-Barr nuclear antigen (EBNA), a positive control protein extract for EBNA (Ext), competition with unlabeled EBNA oligonucleotides, and competition with non-specific oligonucleotides for β-actin. (**B**) Representative EMSA results for cytoplasmic or nuclear (**C**) protein extracts from normoxic testes incubated with biotin-labelled HIF-1 oligonucleotides containing the consensus HRE. Actin lanes contain a molar excess of unlabeled β-actin oligonucleotides to demonstrate the specificity of protein binding to the HIF-1 oligonucleotides.

**Figure 3:**
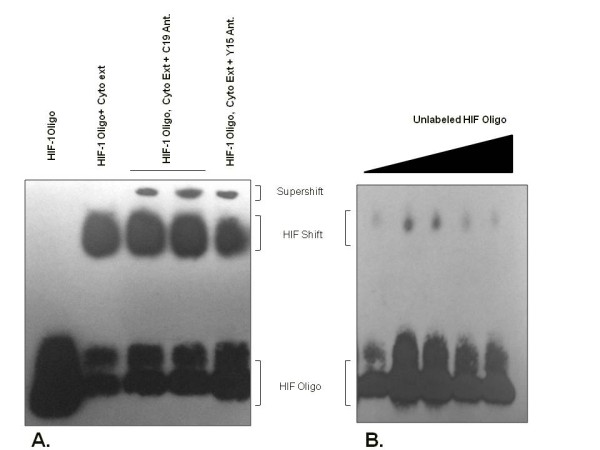
**EMSA supershift and competition assays for testicular HIF**-**1 binding.** (**A**) EMSA results when using cytoplasmic extracts of testicular HIF-1 and the resulting supershifts demonstrated with C19 and Y15 polyclonal antibodies specific for HIF-1α. (**B**) Competition experiments to determine HIF-1 EMSA binding specificity used unlabeled HIF-1 HRE consensus oligonucleotides at increasing concentrations to abrogate HIF-1 oligonucleotide binding by testicular extracts.

The HIF-1 EMSA experiments revealed binding of HIF-1 from cytoplasmic and nuclear extracts from the normoxic testis to biotin-labelled HIF-1 oligonucleotides containing a consensus HRE, demonstrating that testicular HIF-1 is active and capable of binding the HIF HRE (Figure 2). Supershift assays with two different polyclonal antibodies, C19 and Y15, revealed corresponding shifts (Figure 3A). Competition experiments using increasing concentrations of unlabeled HIF-1 oligonucleotides containing the consensus HRE demonstrated the specificity of HIF-1 HRE binding by the testicular extracts (Figure 3B).

**Figure 4:**
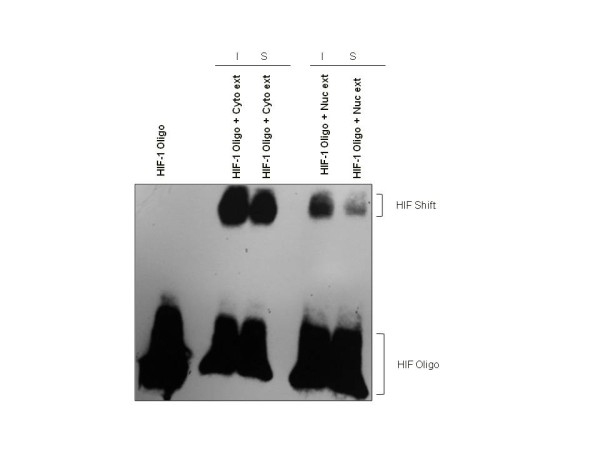
**HIF**-**1 from the normoxic and ischemic testes binds the HIF**-**1 consensus HRE.** Cytoplasmic and nuclear extracts of testicular proteins from normoxic sham-treated (S) and 1 hour ischemic (I) testes were subjected to EMSA to demonstrate HIF-1 binding. Testicular HIF-1 from ischemic and sham-treated testes bound DNA equally.

The EMSA results confirmed the HIF-1 DNA-binding ELISA results, in that both nuclear and cytoplasmic extracts from the testis demonstrated HIF-1 binding in the EMSAs, and there were no quantitative differences detected in HIF-1 binding between extracts from normoxic testes, sham-treated testes and 1h ischemic testes (Figure 4).

### Mcl-1 protein is abundant in the testis and unaffected by ischemia and ischemia/reperfusion

To understand the role of HIF-1 in the testis and molecular pathways involving HIF-1, we were interested in identifying potential target genes for testicular HIF-1. *In silico* analysis of testis genes containing the consensus HRE (5’-RCGTG-3’) identified the induced myeloid leukemia cell differentiation (*Mcl*-*1*) gene as a potential HIF-1 target gene.

**Figure 5:**
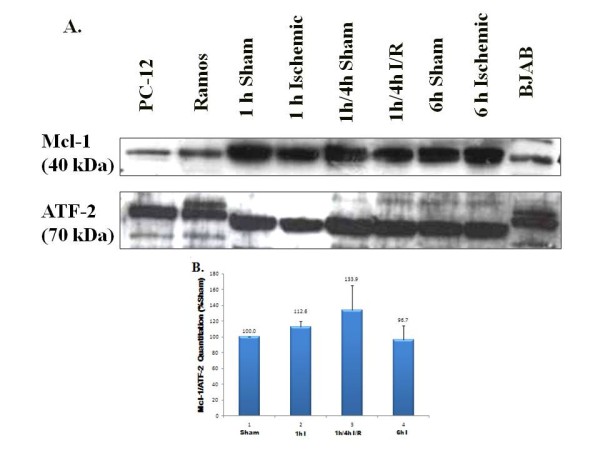
**Mcl**-**1 is abundantly expressed in the testis**, **and Mcl**-**1 levels do not change following ischemia or ischemia**-**reperfusion.** (**A**) Results of Mcl-1 immunoblot analysis of nuclear proteins isolated from experimentally-induced unilaterally ischemic testes (I) and sham-treated testes (S). Aliquots containing 75-μg of nuclear proteins were analyzed from testes subjected to 1 h or 6 h of ischemia, and ischemia and reperfusion (I/R) for 1h/4h. Mcl-1 was detected at ~40kDa. Extracts from the PC-12, Ramos and BJAB cell lines were used as positive controls for detecting Mcl-1. Activating transcription factor-2 (ATF-2) was detected as a loading control as a protein that is constitutively expressed and is unaffected by ischemia. (**B**) Histogram showing Mcl-1 quantitation normalized to ATF-2 as a percentage of the sham controls. No statistically significant differences in the steady state levels of the Mcl-1 protein were observed between the ischemic and normoxic groups (ANOVA, p<0.05, n=3-5).

**Figure 6:**
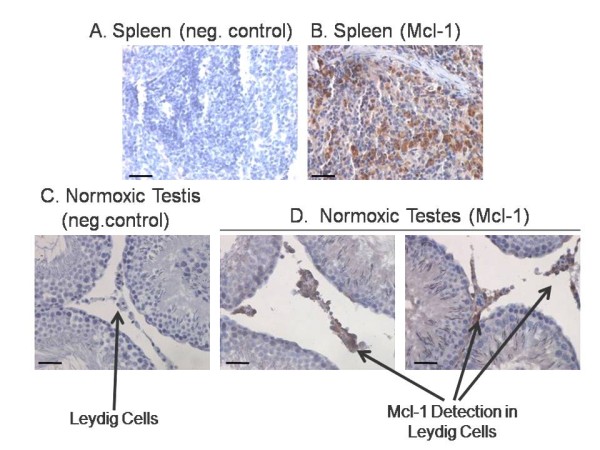
**Mcl**-**1 is present in cultured rat Leydig cells.** Immunoblot analysis of Mc1-1 in cytoplasmic protein extracts from whole, normoxic rat testis (75 μg of protein), and cytoplasmic extracts from ~1 million freshly isolated rat Leydig cells (Fresh L.C.) or Leydig cells cultured for 20 hours under either 5% or 21% oxygen. The large (Mc1-1_L_) and small (Mcl-1_S_) subunits of Mcl-1 are shown. The blot shown here was intentionally over-exposed to reveal low but detectable levels of Mcl subunits in cultured Leydig cells.

Immunoblot analysis of nuclear protein extracts was performed to determine if Mcl-1 is present in the normoxic and ischemic testes. Immunoblots probed with an Mcl-1 primary antibody detected the large, primary subunit of Mcl-1 (40 kDa) in nuclear extracts from sham-treated and ischemic testis (Figure 5A). We were particularly interested in this larger gene product because it is the protein that enhances cell survival by inhibiting apoptosis. Quantitation of Mcl-1 normalized to ATF-2 as a percent of the level in sham control indicated no significant difference in the amount of Mcl-1 protein detected in normoxic and ischemic testis samples (Figure 5B). A smaller, alternatively spliced isoform of Mcl-1, which functions to promote apoptosis, was also found to be present in the testis upon overexposure of blots (see Figure 6) and its expression was unaffected by ischemia (data not shown).

### Immunocytochemistry and immunoblot analyses reveal that Mcl-1 is localized in leydig cells

**Figure 7:**
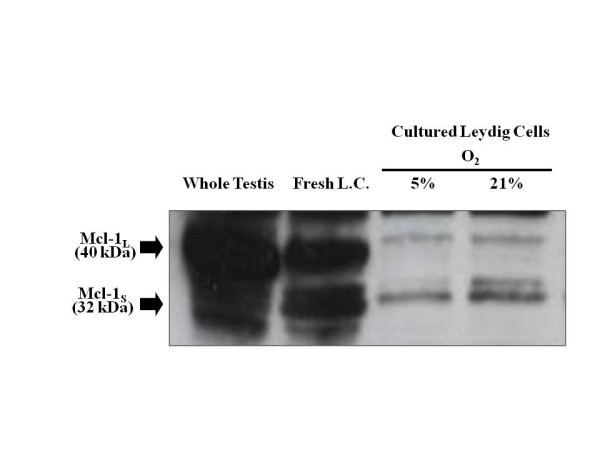
**Immunocytochemical localization of Mcl**-**1 in the normoxic testis.** (**A**) Negative control section from rat spleen incubated with a goat-anti-rabbit HRP secondary antibody in the absence of the Mcl-1 primary antibody (400×). (**B**) Positive control section from a spleen section showing Mcl-1 immunoreactivity (400×). (**C**) Negative control section of normoxic testis incubated with goat-anti-rabbit HRP secondary antibody in the absence of the Mcl-1 primary antibody (400×). (**D**) Normoxic testis section showing Mcl-1 immunoreactivity in Leydig cells indicated by arrows (400×). The sections were counterstained with hematoxylin. Bar, 100 μm.

Once we had determined that Mcl-1 is abundantly expressed in the testis, it was necessary to determine if Mcl-1, like testicular HIF-1, is also expressed by Leydig cells. Spleen was used as a positive control because it is an organ known to express high levels of Mcl-1 (Figure 7A and B). Immunocytochemical analysis detected Mcl-1 protein in the normoxic testis and Mcl-1 was clearly localized to Leydig cells (Figure 7D). Immunoreactivity for Mcl-1 was not observed in negative control sections incubated without primary (Figure 7A and C).

Mcl-1 was also detected in protein extracts isolated from cultured Leydig cells via immunoblot analysis (Figure 7). We previously showed that Leydig cells cultured under 5% or 21% oxygen exhibit down-regulated expression of HIF-1. [12] Interestingly, Mcl-1 expression in Leydig cells cultured under these same conditions also exhibited down-regulated expression. Because we hypothesize that HIF-1 regulates Mcl-1 expression through an HRE in the Mcl-1 promoter, these results provide further support that HIF-1 may regulate Mcl-1 expression in the testis (Figure 6).

### Chromatin immunoprecipitation (ChIP) assays indicate that Mcl-1 is a target for testicular HIF-1

**Figure 8:**
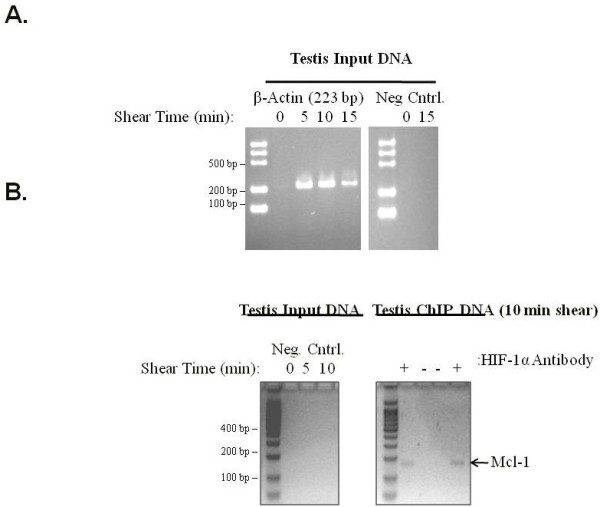
**Chromatin immunoprecipitation enriches for testicular HIF**-**1/chromatin complexes bound to the Mcl**-**1 promoter.** (**A**) Representative example of a ChIP control experiment to determine the optimum shearing time and quality of shearing over time via amplification of β-actin genomic DNA sequences in input DNA from normoxic rat testis. As a negative control, DNA was immunoprecipitated with a β-actin polyclonal antibody for the purpose of determining the specificity of ChIP assays (because β-actin is not a classic DNA-binding protein) and then subjected to PCR. (**B**) ChIP assays to detect HIF-1 binding in normoxic testis demonstrated complexes enriched for Mcl-1 promoter sequences when precipitated using a HIF-1α antibody (+) compared to input DNA that was not immunoprecipitated with the HIF-1α antibody (−). Negative controls in which a nonspecific IgG antibody was used for immunoprecipitation revealed no amplification of Mcl-1 promoter sequences.

ChIP assays were conducted to determine if testicular HIF-1 binds the promoter of Mcl-1 as a HIF-1 target gene *in vivo*. Control experiments demonstrated the effect of shearing time on the quality of chromatin obtained and the specificity of the ChIP assay in pulling down DNA-binding proteins and excluding non-specific, DNA-binding protein complexes using β-actin as a negative control (Figure 8A). Chromatin complexes precipitated with a HIF-1α antibody produced a PCR amplicon when using Mcl-1 primers that was not detectable in PCR assays containing input DNA that was not immunoprecipitated with the HIF-1α antibody (Figure 8B). DNA sequence analysis confirmed that the amplified product detected via PCR with the Mcl-1 primers amplified the Mcl-1 promoter spanning the HRE consensus sequence (data not shown).

## Discussion

Recently, we demonstrated that HIF-1α is the predominant HIF subunit expressed in the rat testis and that Leydig cells are the primary cell type producing HIF-1 in the testis [12]. We also reported that HIF-1α appears to be constitutively present in the normoxic rat testis and is unaffected by ischemia and I/R. In most tissues, HIF-1α is an oxygen-dependent subunit and it has been well established that the hypoxic up-regulation of HIF-1α protein and its rapid degradation under normoxia are controlled through changes in protein stability [22]. The abundance of HIF-1 expressed by normoxic Leydig cells suggests potentially novel roles for HIF-1 in the physiology of the testis.

Investigators have reported normoxic expression of HIF-1α in mesenchymal stem cells [23], cartilage [24], mouse brain and kidney [25], rat kidney [26], and pulmonary and vascular smooth muscle cells [27,28] among other tissues. One goal of the present study was to determine if rat testicular HIF-1 from normoxic and hypoxic testes is capable of binding DNA. Our results from an ELISA-based DNA-binding experiment and from EMSA demonstrated that testicular HIF-1 from normoxic and ischemic tissues bound DNA at equivalent levels, suggesting that testicular HIF-1 is an active transcription factor in both normoxic and ischemic testes. Although we recognize that DNA binding *in vitro* does not necessarily prove that there is transactivation of a promoter by HIF-1 *in vivo*.

Initially, we became interested in studying testicular HIF-1 because we thought that it might play a role in oxygen homeostasis in the testis, particularly under conditions of ischemia and ischemia-reperfusion, such as during testicular torsion. Ischemia and ischemia-reperfusion of the testis result in germ cell apoptosis. However, after we localized HIF-1 to Leydig cells we hypothesized that one role of HIF-1 may be to activate antiapoptotic target genes to protect Leydig cells from apoptosis during ischemia and ischemia-reperfusion. HIF-1 may also play other important roles in Leydig cell physiology. For example, in the mouse testis, HIF-1α regulates the expression of the 3β-hydroxysteroid dehydrogenase type I (*Hsd3b1*) gene in Leydig cells [14], and up-regulated expression of HIF-1α has been reported in experimentally-induced varicoceles in the rat testis [29].

An emerging body of research has also demonstrated that HIF-1-mediated pathways are involved in inflammatory responses [30,31]. Recent work in our laboratory has shown that testicular HIF-1 expression is up-regulated by LPS-induced inflammation of the testis (unpublished observations, Patel, D., Dugan, C., Karpodinis, M, Fasano, G. M., and Palladino, M.A. Up-regulation of testicular hypoxia-inducible factor-1α following *E*.*coli* and *P*. *aeruginosa* lipopolysaccharide-induced inflammation. Manuscript under review).

For a variety of reasons, the testis has been described as a hypoxic tissue [32]. Studies designed to measure the physiological oxygen tension in the testis indicate that testicular pO_2_ levels are likely low, bordering on hypoxia [16,17]. As we have reported previously [12], the low oxygen tension in this tissue under conditions that are generally considered to correspond to normoxic physiology is likely the reason that HIF-1 is highly expressed in the “normoxic” testis and is apparently unregulated by I and I/R, in contrast to what is observed in other tissues. Therefore, the functional significance of constitutive expression of testicular HIF-1α in the normoxic testis is of significant interest to us.

Once we established that in rats testicular HIF-1 binds DNA equally under normoxic and ischemic conditions, we turned our attention to identifying potential HIF-1 target genes in the testis. *In silico* analysis of testis genes containing a consensus hypoxia response element (HRE, 5’-RCGTG-3’) identified the induced myeloid cell leukemia differentiation (*Mcl*-*1*) gene as a potential HIF-1 target gene. Immunoblot analysis to detect MCL-1 demonstrated that MCL-1 protein is highly expressed in the normoxic and ischemic testis and is localized to Leydig cells, presenting a pattern that mimics the distribution of HIF-1.

Through ChIP assays using anti-HIF-1 antibodies we isolated chromatin complexes enriched for Mcl-1 promoter DNA sequences and sequence analysis confirmed that the HIF-1 complexes contained the HRE of the Mcl-1 promoter. Mcl-1 is member of the B-cell lymphoma family of anti-apoptotic proteins [33] and is an active target in cancer research [34]. Examination of the antiapoptotic effects of HIF-1 in endothelial cells [35] has shown that the antiapoptotic roles of HIF-1 are mediated via regulating the expression of Mcl-1 [36]. Recently, Iqbal et al. demonstrated that Mcl-1 expression is up-regulated by platelet derived growth factor (PDGF) in a HIF-1-dependent manner in prostate cancer cells and that pharmacological inhibition of the PDGF receptor decreases Mcl-1 levels and induces apoptosis in metastatic prostate cancer cells [37].

Here, we propose that the co-localization of HIF-1 and Mcl-1 observed in Leydig cells, showing a coordinated pattern of expression, together with the results of our experiments using a HIF-1 antibody in ChIP analysis, which identified the HRE in the *Mcl1* promoter in HIF-1-precipitated complexes, provides evidence that testicular HIF-1 binds to *Mcl1* as a target gene. We recognize that these results alone do not definitively demonstrate a functional or direct role for HIF-1 or Mcl-1 in the testis. However, we previously reported that Leydig cells cultured under 5% or 21% oxygen showed down-regulated expression of HIF-1 [12]. In the current study, Mcl-1 expression in Leydig cells cultured under these same conditions was also diminished, suggesting that HIF-1 expression correlates well with Mcl-1 expression in a coordinated fashion, though we did not determine if an increase in Leydig cell apoptosis resulted from down-regulated expression of Mcl-1 in these *in vitro* experiments.

Pharmacological studies are currently underway in our laboratory to determine the physiological significance of the inhibition of HIF-1 and/or Mcl-1 in Leydig cell biology in normoxic and hypoxic testes. We are also investigating the molecular mechanisms involved in stabilizing testicular HIF-1 in the normal testis *in vivo* under conditions that are presumed to be normoxic.

## Conclusions

In conclusion, we have demonstrated that testicular HIF-1 from normoxic and ischemic rat testes binds DNA, suggesting potentially novel roles for HIF-1 and Leydig cells in regulating the cellular and molecular responses to oxygen in the testis. We have provided evidence that Mcl-1 is a target gene of HIF-1. Further exploration of this interaction will be essential for exploring cellular and molecular responses to ischemia and hypoxia in the testis to advance our understanding of the role of HIF-1 in Leydig cells and the pathophysiologic effects of hypoxia and other conditions affecting the HIF-1 pathway in the testis.

## Abbreviations

I: Ischemia; I/R: Ischemia-reperfusion; HIF-1: Hypoxia-inducible factor-1; HRE: Hypoxia response element; Mcl-1: Myeloid cell leukemia-1; ELISA: Enzyme-linked immunosorbent assay; ChIP: Chromatin immunoprecipitation analysis; EMSA: Electrophoretic mobility shift assay; AVMA: American Veterinary Medical Association; DTT: Dithiothreitol; ANOVA: Analysis of Variance; EBNA: Epstein-Barr Nuclear Antigen; TdT: Terminal deoxynucleotide transferase; SDS-PAGE: Sodium dodecyl sulfate polyacrylamide gel electrophoresis; HRP: Horseradish peroxidase; PBS: Phosphate-buffered saline; PMSF: Phenylmethylsulfonylfluoride; PIC: Protease inhibitor cocktail; PCR: Polymerase chain reaction; kDa: Kilodalton; NIH: National Institutes of Health.

## Competing interests

The authors declare that they have no competing interests.

## Authors' contributions

MAP conceived of the study, participated in its design and coordination and drafted the manuscript. AS carried out ELISA DNA-binding experiments and drafted appropriate portions of the manuscript. RT and JH carried out Mcl-1 western blot experiments, Mcl-1 immunocytochemistry and ChIP analysis and drafted appropriate portions of the manuscript. CD and MC performed EMSA experiments and drafted appropriate portions of the manuscript. All authors read the draft manuscript and approved the final manuscript.

## Authors’ information

The authors wish to disclose that functional studies on HIF-1 are currently underway in the laboratory using pharmacologic agents to disrupt HIF-1, Mcl-1 and other aspects of the HIF-1 pathway. A limitation of the current manuscript is that it does not provide data from functional studies. However, the PI maintains a small laboratory staffed with undergraduate students and the time necessary to complete the functional studies and include them in the current manuscript would delay publication for several years. The author’s would prefer to publish the current study as soon as possible to share these results with the community of researchers that have been interested in this work. The following co-authors were Monmouth University students when the studies described in this manuscript were carried out and they have all graduated. AS is currently a medical student at Drexel University College of Medicine. RT is currently a student in the physician’s assistant program at Seton Hall University. JH is currently a nursing student at Brookdale College, CD is currently employed by Church & Dwight, and MK is currently employed by Laureate Pharm.
